# Finite-element analysis of microwave scattering from a three-dimensional human head model for brain stroke detection

**DOI:** 10.1098/rsos.180319

**Published:** 2018-07-11

**Authors:** Awais Munawar Qureshi, Zartasha Mustansar, Samah Mustafa

**Affiliations:** 1 Research Center for Modeling and Simulation (RCMS), National University of Sciences and Technology (NUST), H-12 Islamabad 44000, Pakistan; 2 College of Engineering, Salahaddin University, Erbil 44002, Iraq

**Keywords:** brain stroke, finite-element method, forward problem, human head model, inverse problem, microwave imaging

## Abstract

In this paper, a detailed analysis of microwave (MW) scattering from a three-dimensional (3D) anthropomorphic human head model is presented. It is the first time that the finite-element method (FEM) has been deployed to study the MW scattering phenomenon of a 3D realistic head model for brain stroke detection. A major contribution of this paper is to add anatomically more realistic details to the human head model compared with the literature available to date. Using the MRI database, a 3D numerical head model was developed and segmented into 21 different types through a novel tissue-mapping scheme and a mixed-model approach. The heterogeneous and frequency-dispersive dielectric properties were assigned to brain tissues using the same mapping technique. To mimic the simulation set-up, an eight-elements antenna array around the head model was designed using dipole antennae. Two types of brain stroke (haemorrhagic and ischaemic) at various locations inside the head model were then analysed for possible detection and classification. The transmitted and backscattered signals were calculated by finding out the solution of the Helmholtz wave equation in the frequency domain using the FEM. FE mesh convergence analysis for electric field values and comparison between different types of iterative solver were also performed to obtain error-free results in minimal computational time. At the end, specific absorption rate analysis was conducted to examine the ionization effects of MW signals to a 3D human head model. Through computer simulations, it is foreseen that MW imaging may efficiently be exploited to locate and differentiate two types of brain stroke by detecting abnormal tissues’ dielectric properties. A significant contrast between electric field values of the normal and stroke-affected brain tissues was observed at the stroke location. This is a step towards generating MW scattering information for the development of an efficient image reconstruction algorithm.

## 1. Introduction

Over the past few years, the increase in brain stroke incidences is alarming [1,2]. It causes an intermittent supply of blood to critical areas of the brain which disturbs the continuous flow of oxygen and nutrients to brain tissues. This phenomenon not only results in the dysfunction of human brain functioning but may cause death in many cases. Brain stroke is classified into two major categories: haemorrhagic and ischaemic [3]. Both share some common symptoms, e.g. sudden weakness of body parts, severe headache leading to unconsciousness, trouble with speaking and difficulty with swallowing. However, the treatment of each type involves an exclusive and timely medication from the onset of stroke symptoms [4].

To investigate the presence of brain stroke, several imaging techniques are used including computed tomography (CT), magnetic resonance imaging (MRI) and positron emission tomography (PET). These techniques can efficiently provide high-resolution images of the human brain but they have certain constraints associated with them [5–7]. This situation necessitates an alternate imaging technique which can offer a cost-effective, portable and safe imaging option for reliable and rapid brain stroke diagnostics. In recent years, microwave tomography (MWT) using MW signals has emerged as a promising brain-imaging modality. MWT possesses non-ionizing and non-invasive features while offering all of the above advantages as discussed.

**Figure 1.**
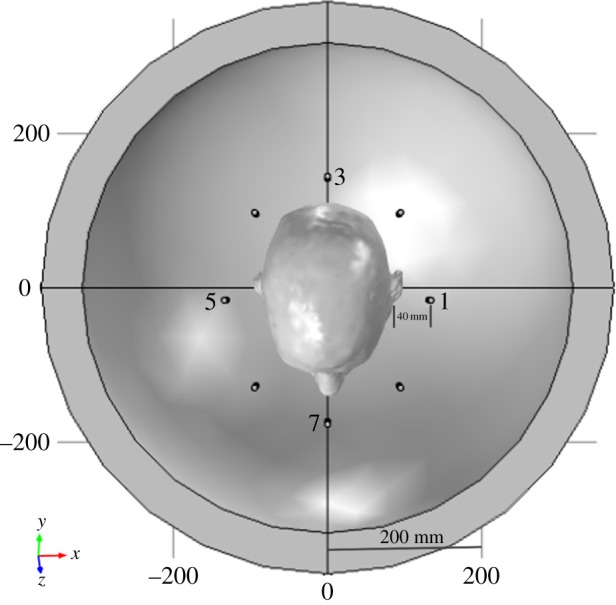
Layout of a three-dimensional microwave head-imaging system.

In microwave imaging (MWI), reliable images of the object-of-interest are constructed by measuring and processing the transmitted and backscattered signals from the target. MWI takes advantage of a significant contrast between dielectric properties of the target and its surroundings [8]. In active MWI, the two main approaches in practice are the Confocal Radar Technique and Classical Inverse Scattering (also known as MWT) [9,10]. Microwave head-imaging systems for brain stroke diagnostics employ one of these techniques. In the case of an emergency, particularly in rural areas, these systems can efficiently supplement the existing brain-imaging modalities (CT, MRI or PET). They can be easily deployed at emergency rooms or carried out in first-response ambulances. MW signals in the frequency range of 0.5–4.5 GHz and the 0–20 dBm power level make these systems suitable for continuous brain monitoring [11]. Figure 1 shows the layout of a conventional three-dimensional (3D) MW head-imaging set-up.

In this study, different aspects of microwave scattering from a 3D human head model have been explored for brain stroke analysis. The MW scattering behaviour of a human head is explained through image-based finite-element (FE) simulations. Image-based modelling is a new approach to perform the realistic simulation of scientific problems in a virtual reality [12,13]. A 3D anatomically realistic head model has been developed using an available stack of MRI slices. The frequency-dependent dielectric properties of 21 tissue types are assigned to the head model using a novel tissue-mapping scheme and a mixed-model approach. This 3D numerical head model can be effectively used during the simulation of various biomedical engineering applications. FE mesh convergence and iterative solvers comparison analysis are also conducted to attain reliable results in minimal computational time. The next section presents a brief overview of the literature in the area of MW head imaging, followed by the research methodology, results and discussion, and conclusion sections.

## 2. Background

Initial studies to investigate the performance of the MW head-imaging technique for brain stroke detection involved geometrically simplified human head models. These two-dimensional (2D)/3D geometric models incorporated the major layers of brain tissue to mimic the behaviour of a human head. A preliminary study was conducted by Semenov & Corfield [14] in 2007. The authors evaluated the performance and practicability of MW head imaging for brain stroke diagnostics. They performed computer simulations on a concentric circle-based 2D head model comprising five basic layers of brain tissue. An ischaemic brain stroke with a radius of 20 mm was emulated in a layer of white matter (WM). A MW signal was transmitted at the 20 dBm power level in the 0.5–2.0 GHz frequency range. A nonlinear Newton reconstruction approach was adopted to construct reliable images. The results were very promising and supported the exploitation of the MW scattering phenomenon for brain stroke detection and progression monitoring.

It was in 2010 that Ireland & Bialkowski [15] for the first time involved a 3D realistic human head model to perform the MW scattering analysis for brain stroke detection. A cylindrical shape haemorrhagic stroke of diameter 30 mm was emulated in the Zubal head phantom [16]. The finite-difference time-domain (FDTD) numerical method was applied to solve this 3D MW scattering problem. The amplitude and phase differences in scattered electric field (E-Field) signals at antenna locations were used to indicate the presence of brain stroke. Building on their initial results, in 2011 Ireland & Bialkowski developed a 2D image reconstruction algorithm using scattered E-Field information from a 2D MRI slice head model [17]. The inverse algorithm was based on common background reflections cancellation, the confocal delay-and-sum (DAS) approach and Fermat's principle [18–21]. A Gaussian pulse signal in the frequency range of 0.5–2.0 GHz at a 20 dB signal-to-noise ratio was used in the simulations.

In 2010, Zakaria A *et al.* [22] followed a finite-element method (FEM)–contrast source inversion (CSI)—to develop a 2D image reconstruction algorithm for haemorrhagic stroke detection. A 2D human head was modelled using five concentric ellipses, each representing a major layer of brain tissue. An array of 32 evenly spaced point transceivers operating in a multi-static mode at the 1 GHz frequency was used. In 2012, Scapaticci *et al.* [23] devised the design guidelines for an effective head-imaging system. The objective was to achieve a maximum penetration of MW signals into the human head (frequency: 0.6–1.5 GHz, matching medium: ε_mm_ = 40, *σ*_mm_ = 0.01 S m^−1^). The design analysis was based on transmission line (TL) theory. A human head was modelled as five basic layers of brain tissue with distinct characteristic impedance. The authors validated these guidelines through 2D computer simulations using the Zubal head phantom slices with an emulated ischaemic stroke. The method of moments (MoM) numerical technique was applied to calculate scattered E-Field values and the imaging strategy was based on a modified linear sampling method.

Significant research contributions have been made in the field of microwave head imaging from 2013 onwards. Jalilvand *et al.* [24] formulated an analytical model for brain stroke analysis based on an ultra-wide band (UWB) radar approach. A 2D planar head model was incorporated using five layers of brain tissue and an added layer of blood emulating the haemorrhagic stroke. Both the transmission and reflection scenarios were investigated using UWB signals (up to 10 GHz) with an effective power of 0 dBm at the first layer. The authors also implemented an FDTD–iterative Gauss-Newton approach to present a quantitative analysis of MWT for haemorrhagic stroke detection [25]. A 2D realistic head model surrounded by 24 point sources was used. Later on, a helmet-based MW head-imaging system for brain stroke localization and classification was designed by Fhager *et al.* [26]. An array of triangular patch antennae (0.1–3.0 GHz) was mounted inside the helmet with matching medium-filled plastic bags for head size adjustment. Two types of stroke were differentiated through a statistical classifier algorithm. Priyadarshini and Rajkumar [27] applied a FEM–CSI methodology on a four-layered ellipsoid head model to explore the MW scattering phenomenon for brain stroke detection of both types. The head model was surrounded by an array of eight dipole antennae, each operating in multi-static mode at the 1 GHz frequency.

The medical imaging research group at the University of Queensland (Australia) has contributed remarkably in the area of MW head imaging for brain stroke detection [11,28]. Primarily, this research group followed a radar approach by implementing the confocal DAS algorithm and Fermat's principle. Noise in the received signals from background reflections was removed by applying a preprocessing technique [29]. The designed head-imaging system comprised an array of 16 tapered slot antennae (1–4 GHz) installed on an adjustable platform. A human head phantom comprising major types of brain tissue was also fabricated [30]. Later, in 2014, a portable head-imaging system for brain injuries and haemorrhagic stroke detection was proposed [31,32]. It operated in a virtual array monostatic radar mode using a unidirectional antenna and a transceiver. The authors also investigated the feasibility of deploying a similar system to classify two types of brain stroke. Computer simulations were performed involving a SAM head model having average dielectric properties of brain tissues. A stroke of both types and different shapes was emulated at various locations inside the head model [33,34]. The first approach compared the reflection coefficients (S_11_) of a pair of antennae located symmetrically around the head, whereas the second approach used the reflection phase information.

In 2016, Mobashsher *et al.* [35] conducted a design analysis and the experimental validation of a portable head-imaging system (1–3 GHz) following a monostatic radar approach. An improved back-projection algorithm was also developed to detect the presence of an intracranial haemorrhage (1 cm^3^) inside the fabricated head phantom [36]. Later on, Zamani *et al.* [37] presented a fast multi-static MWI algorithm for the detection of brain injuries based on frequency-domain analysis. The algorithm was validated through numerical simulations and a radar-based head-imaging set-up. In the same year, Dilman *et al.* [38] demonstrated the capability of a MoM–CSI methodology to investigate the feasibility of MWT for haemorrhagic stroke detection. A single head slice illuminated by 36 line sources was used. This study also incorporated the effects of different background media and an Additive White Gaussian Noise. Afterwards, the authors followed a differential MWI approach to monitor the evolution of a haemorrhagic area over two time frames [39].

In 2017, Bisio *et al.* [40] implemented an MoM–iterative Gauss-Newton scheme to evaluate the performance of MWT for haemorrhagic stroke detection in a 2D head slice. The authors also investigated the suitable operating conditions for a MWI system and the effects of background medium permittivity on image reconstruction results. In the same year, Ricci *et al.* [41] combined the principal component analysis (PCA)-based artefact removal technique and a modified-DAS beam-forming algorithm for haemorrhagic stroke detection in a simplified 3D SAM head model. Recently, Hopfer *et al.* [42] developed an electromagnetic tomography (EMT) brain scanner by designing a spherical chamber installed with 177 waveguide antennae in an eight-rings configuration. Two types of brain stroke were analysed using different numerical solvers (FEM, FDTD), a gradient-based inverse algorithm and the experimental set-up. The results were satisfactory but a relatively simple 3D head phantom was used.

In our earlier studies, we investigated the feasibility of EMT for brain stroke diagnostics using MW signals [43]. FEM-based frequency-domain analysis was performed using a 3D ellipsoid head model and an array of half-wave dipole antennas. We also validated our simulation results through an analytical analysis. Later on, we extended our research on a 2D realistic head model to present a MW scattering comparison analysis for the detection and differentiation of different types of brain stroke [44]. The head model was surrounded by an array of point current sources, each operating in a sequential mode. We also conducted a level of details analysis of MW scattering from different complexity head models. The aim was to highlight the significance of incorporating realistic details into head models during computer simulations for brain stroke analysis [45].

In our present research, a detailed analysis of the MW scattering phenomenon exhibited by a human head model is presented in 3D. An anthropomorphic 3D head model is created using an MRI database. Frequency-dispersive dielectric properties are assigned to brain tissues by implementing a novel tissue-mapping scheme along with a mixed-model approach. FE analysis is used for the localization and classification of both types of brain stroke, emulated at various locations inside the head model. In addition, FE mesh convergence and iterative solvers comparison analysis is also performed to find out a solution of the subject 3D MW scattering problem in an error-free and time-efficient manner. There is no dedicated study available in the literature to date which presents this comparison analysis in the area of MW head imaging.

## 3. Material and methods

This section describes the research methodology used in this study in a stepwise manner. Initially, we developed an anatomically more realistic and structurally detailed human head model. This 3D head model was then assigned frequency-dispersive dielectric properties of brain tissues. Two types of brain stroke were emulated at different depths inside the head model. An antenna array was designed in the simulation environment to perform the MW scattering analysis of a 3D head model. The information established from mesh convergence and computational time analysis is fully used during the next section's simulations so as to determine the feasibility of MWT for brain stroke diagnostics.

### 3.1. Human head modelling

**Figure 2.**
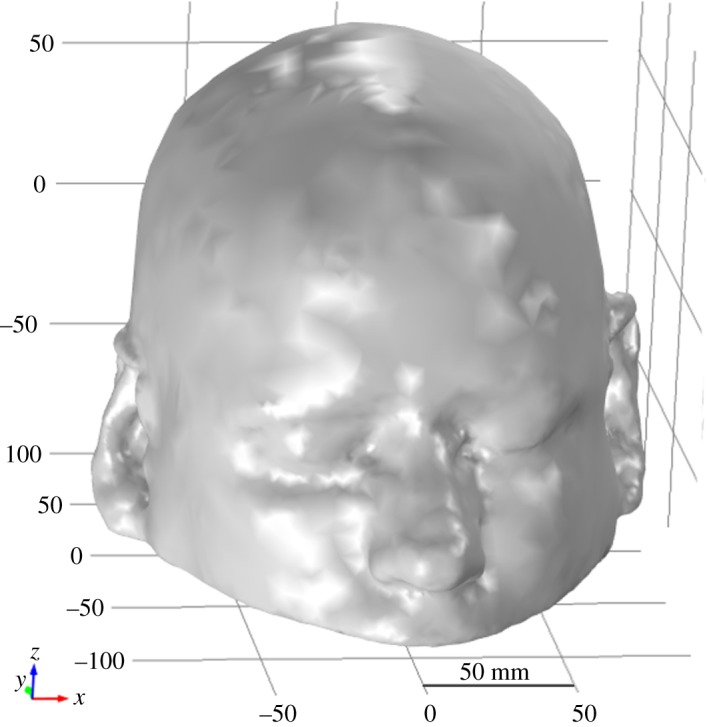
A three-dimensional view of the Zubal head phantom shell geometry.

The Zubal head phantom MRI database is used in this study for the geometrical construction of a 3D numerical head model [46]. This dataset comprises 128 slices, where each slice contains 256 × 256 cubical elements of voxel size 1.1 × 1.1 × 1.4 mm. These slices were imported into the Simpleware ScanIP image processing suite and single mask segmentation was applied. A 3D tetrahedral mesh was exported from the Simpleware suite in order to create the Zubal head phantom shell geometry in the electromagnetic (EM) simulation environment (figure 2). The Zubal head phantom was preferred in this study because it offered us an opportunity to compare our simulation results with earlier research studies involving the complete Zubal head phantom. Previous studies applied different tissue-mapping schemes as well as the simulation approaches [15,35,36,47–49]. In this study, frequency-dispersive dielectric properties were assigned to different types of head tissues by importing the voxel assignment tissues' property files in the simulation environment.

### 3.2. Dielectric properties assignment

A novel dielectric properties assignment approach was implemented in the Matlab software to prepare tissues' property files. The dielectric properties were assigned to each voxel location in the files using the phantom developer's tissues ID list, an innovative tissue-mapping scheme and two frequency-dependent closed-form equations. The first equation is a fourth-order Cole-Cole model (equation (3.1)), developed for the assignment of dielectric properties to over 30 types of human body tissues across the 10 Hz to 20 GHz frequency range [50–54]. The second equation is computationally less intensive and more accurate, explicitly formulated for the assignment of dielectric properties to 17 types of human head tissues only [48]. It is termed as the fourth-order Debye model (equation (3.2)), valid across the 0.1–3 GHz MW frequency. Both closed-form models' parameter values were extracted from their relevant research papers. In these equations, $\epsilon_{r}^{\prime }(\omega )$ denotes the complex relative permittivity as a function of angular frequency (*ω*) and comprises the frequency-dependent relative permittivity *ε_r_(ω)* and electrical conductivity *σ(ω)* parts.

3.1 $$\epsilon_{r}^{\prime }(\omega )=\epsilon_{\infty }+\overset{4}{\underset{m=1}{\sum }}\frac{\Delta \epsilon_{m}}{1+{\left(j\omega \tau_{m}\right)}^{1-\alpha_{m}}}+\frac{\sigma_{i}}{j\omega \epsilon_{0}}$$

and

3.2 $$\epsilon_{r}^{\prime }(\omega )=\epsilon_{\infty }+\overset{4}{\underset{i=1}{\sum }}\frac{\Delta \epsilon_{i}}{1+j\omega \tau_{i}}+\frac{\sigma_{s}}{j\omega \epsilon_{0}}.$$

**Figure 3.**
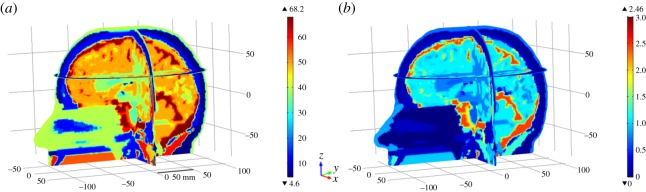
Dielectric profile of the 3D Zubal head model at the 1 GHz frequency: (*a*) relative permittivity and (*b*) electrical conductivity.

While preparing the voxel assignment tissues' property files, primarily we used the fourth-order Debye model to assign the dielectric properties to 56 types of brain tissues, whereas the rest of the four types used the fourth-order Cole-Cole model. In this way, we mapped 60 tissue types of the complete Zubal head model into 21 types by using a mixed-model approach involving our novel tissue-mapping scheme. The aim was to assign tissues' dielectric properties in an anatomically and spatially more accurate fashion, while keeping the execution time of the Matlab programmed function to a minimum. The dielectric properties of mapped head tissues at the 1 GHz frequency along with model type are listed in table 1. The dielectric profile of the Zubal head model at the 1 GHz frequency is shown in figure 3.

**Table 1.** Dielectric properties of the mapped head tissues at the 1 GHz frequency.

s. no.	organ/tissue type	relative perm. (ε_r_)	conductivity *σ* ( S m^−1^)	model type
1	air/free space	1	0	fourth-order Debye
2	skin (dry)	39.6666	0.7062	
3	skin (wet)	47.7684	0.9314	
4	fat	4.602	0.0535	
5	bone, cortical	12.6348	0.1831	
6	bone, cancellous	18.7217	0.328	
7	white matter (WM)	37.8741	0.7477	
8	grey matter (GM)	49.7739	1.0916	
9	blood	55.5956	1.8885	
10	cerebrospinal fluid (CSF)	68.1756	2.4603	
11	dura	48.0121	1.0189	
12	bone marrow	5.1292	0.0584	
13	cerebellum	49.4544	1.0968	
14	spinal cord (nerve)	33.1778	0.6322	
15	eye tissue (sclera)	54.6089	1.2415	
16	cartilage	40.2931	0.8736	
17	muscle, parallel	60.3305	1.2905	
18	stomach	64.7973	1.2316	fourth-order Cole-Cole
19	tongue	55.017	0.9751	
20	trachea	41.7785	0.8023	
21	eye lens (nucleus)	35.6667	0.5118	

We also performed a careful analysis of the frequency range (0.5–2.0 GHz) defined in the literature for MW head-imaging applications [14,17,23,40]. Initially, we conducted our simulations on simpler head models with frequency-dispersive dielectric properties of brain tissues. However, in this study the incorporated frequency-dependent closed-form models are evaluated at 1 GHz only. This is because 1 GHz is the most appropriate frequency for a MW head-imaging system, suggested by the previously referenced literature [14,23,25,27,35,38,40,42] and the evaluations we made during our research [43–45]. This frequency allows a good penetration of MW signals into a human head, while providing a reasonable spatial resolution of brain images. Therefore, we have presented our 3D human head simulation analysis at the same frequency.

### 3.3. Brain stroke emulation

**Figure 4.**
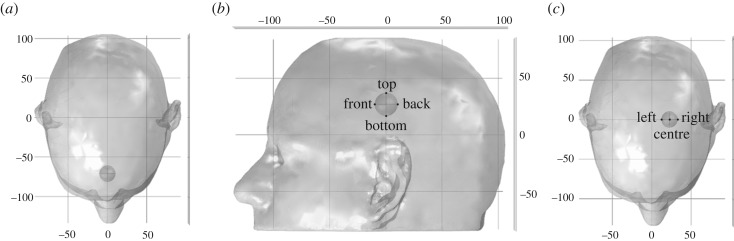
Brain stroke emulation: (*a*) front location-1, (*b*) centre location-2 and (*c*) side location-3.

A sphere-shaped stroke with a radius of 10 mm was emulated at three different locations in slice 45 of the Zubal head model (frontal, central and lateral) [33,34,41]. Figure 4 shows the positions of an emulated stroke with E-Field evaluation points inside the Zubal head model.

(i) The stroke sphere was centred at a shallow location (0 mm, −70 mm, 26.6 mm), where the major part of the stroke lay in the grey matter (GM) region of the frontal area (figure 4*a*).
(ii) The stroke sphere was centred at a deep location (0 mm, 0 mm, 26.6 mm), where the stroke area intersected both the GM and WM regions of the central area (figure 4*b*).
(iii) The stroke sphere was again centred at a shallow location (22 mm, 0 mm, 26.6 mm); however, the major part of the stroke was in the WM region of the lateral area towards the right (figure 4*c*).

**Figure 5.**
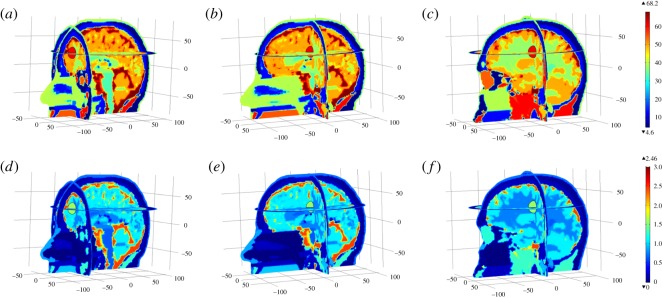
Dielectric profile of the haemorrhagic Zubal head model at the 1 GHz frequency: (*a*) relative permittivity at the front location, (*b*) relative permittivity at the centre location, (*c*) relative permittivity at the side location, (*d*) conductivity at the front location, (*e*) conductivity at the centre location and (*f*) conductivity at the side location.

**Figure 6.**
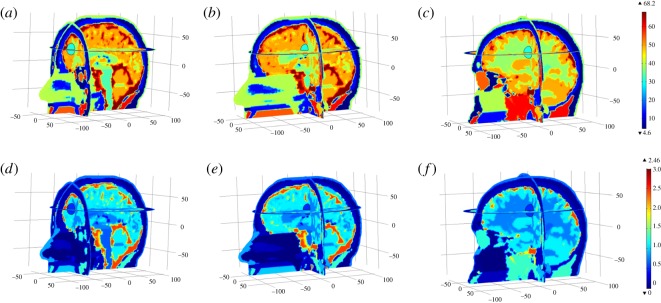
Dielectric profile of the ischaemic Zubal head model at the 1 GHz frequency: (*a*) relative permittivity at the front location, (*b*) relative permittivity at the centre location, (*c*) relative permittivity at the side location, (*d*) conductivity at the front location, (*e*) conductivity at the centre location and (*f*) conductivity at the side location.

The effects of two main types of brain stroke on MW scattering behaviour of the Zubal head model were analysed at the above three locations. At the 1 GHz frequency, a haemorrhagic stroke was assigned with bleeding dielectric properties (ε_r_ = 61.0650, *σ* = 1.5829 S m^−1^) [17,22,27,33–35,38,40], whereas an ischaemic stroke was assigned (ε_r_ = 30, *σ* = 0.5 S m^−1^) dielectric properties, around 10% less than the WM properties (ε_r_ = 37.8741, *σ* = 0.7477 S m^−1^) [14,23,33,34,42]. Figures 5 and 6 provide a 3D dielectric profile map of the Zubal head model, emulated with two types of stroke at various locations inside the brain.

### 3.4. Antenna array designing

**Figure 7.**
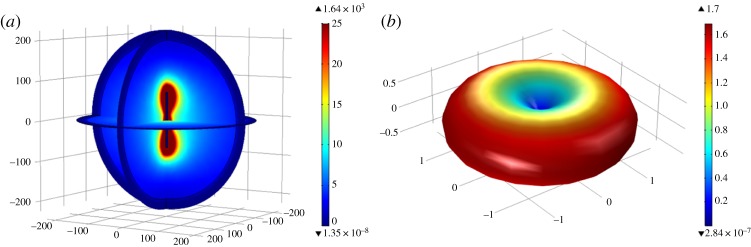
Dipole antenna patterns at the 1 GHz frequency: (*a*) electric field norm (V m^−1^) and (*b*) far-field gain.

**Figure 8.**
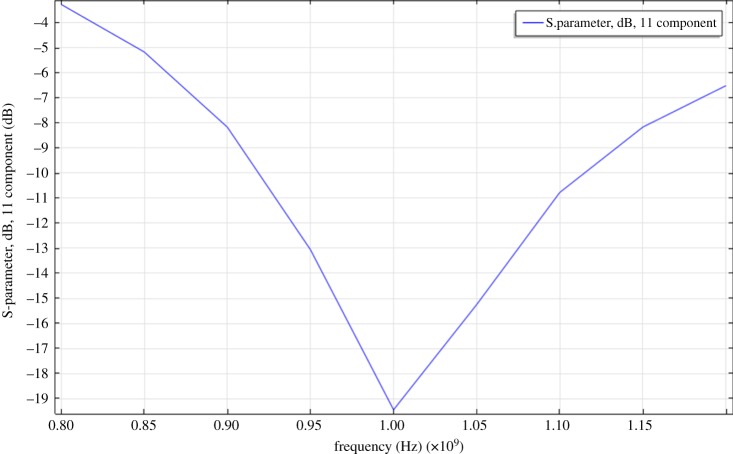
A dipole antenna's reflection coefficient (*S*_11_) frequency sweep plot.

To transmit and receive MW signals around the Zubal head model, an eight-elements antenna array was designed using half-wave dipole antennae. The dipole antenna was modelled to achieve a centre frequency ( *f*_c_) of 1 GHz with a reflection coefficient (*S*_11_) < −10 dB in a free-space medium. The design parameters of a simulated dipole antenna are listed in table 2. Figure 7 shows the E-field norm (E-Norm) and far-field gain patterns of a dipole antenna transmitting at the 1 GHz frequency with 6.6 mW power. Figure 8 plots the reflection coefficient (*S*_11_) of a dipole antenna across the 0.8–1.2 GHz frequency band. It has been observed that the designed dipole antenna has a desired omnidirectional far-field pattern with a 1.7 gain factor. It can efficiently operate in the frequency range of 0.92–1.12 GHz with a reflection coefficient less than −10 dB in free space. In this study, the dipole antenna was designed for a 1 GHz centre frequency because this value is reported as the most suitable frequency by the literature to date for the design of an effective MW head-imaging system [14,23,25,27,35,38,40,42]. Therefore, subsequent simulations have been performed at the same frequency.

**Table 2.** Design parameters of the dipole antenna with a centre frequency of 1 GHz.

s. no.	parameter	symbol/formula	value
1	centre frequency	*f*_c_	1 × 10^9^ Hz
2	speed of light	*c*	3 × 10^8^ m s^−1^
3	wavelength	*λ* = *c*/*f*	0.3 m
4	resonance factor	*R*_f_	0.46
5	antenna length	*L* = *R*_f_ × *λ*	138 mm
6	arm length	*L*_a_ = *L*/2	69 mm
7	antenna radius	*L*_a_/20	3.45 mm
8	gap size	*L*_a_/100	0.69 mm
9	bandwidth	*f*_l_ to *f*_h_	0.92 to 1.12 GHz
10	reflection coefficient	*S*_11_	<−10 dB
11	input voltage	*V*_0_	1 V
12	input impedance	*Z*_0_	78 Ω
13	transmitted power	*P*_t_	6.6 mW
14	far-field gain	*G*	1.7

**Figure 9.**
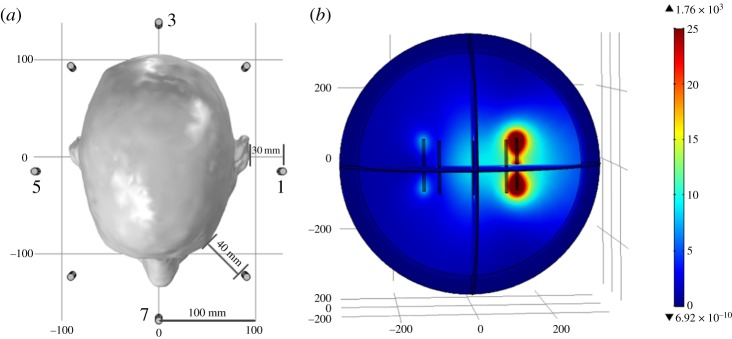
Eight-elements dipole antenna array: (*a*) elliptical array configuration and (*b*) spatial distribution of E-Norm (V m^−1^) for dipole antenna-1 at the 1 GHz frequency.

The eight dipole antennae were arranged in an elliptical manner around the Zubal head model. Each antenna was placed at a distance of 3–4 cm from the side of the head model, with equal distance from the adjacent one [14,15,25,27]. The antenna array followed a multi-static radar approach, in which the elements operated in a sequential way, and once one antenna was transmitting, the rest were in the receiving mode. In our simulations, each dipole antenna operated at the 1 GHz frequency with an input voltage of 1 V at 75 Ω impedance. Figure 9 shows an elliptical array configuration of eight dipole antennae and the spatial distribution of E-Norm for a dipole antenna placed at location-1 in the array. The transmission coefficient (*S*_21_) was measured to investigate the mutual coupling between an adjacent pair of dipole antennae in the array. The *S*_21_ value was calculated as −9.77 dB at the 1 GHz frequency, which can be further reduced by designing an array of directional antennae. In this study, we have deployed an eight-elements antenna array to investigate the difference between the MW scattering behaviour of a normal and stroke-affected head model at various locations inside the brain for a free-space medium [27,33,37,41]. In future, based upon the design requirements of our imaging set-up and the reconstruction performance of our inverse algorithm, we will add more antennae and a suitable coupling medium around the head model, in order to enhance the head coverage and improve the imaging resolution.

### 3.5. Forward problem solution

**Figure 10.**
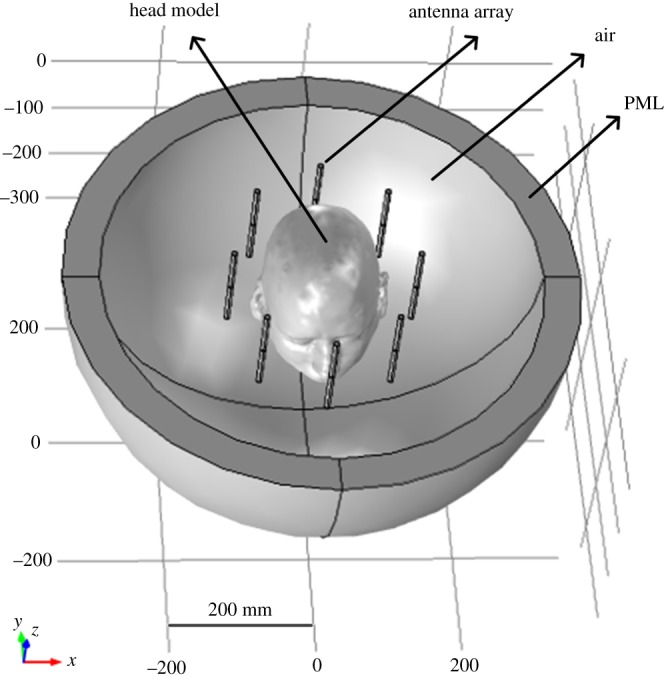
Microwave head-imaging simulation set-up for the forward problem.

To determine the design parameters and evaluate the performance of a MW head-imaging set-up, a virtual model is built in an EM simulation platform. Thereafter, simulation results are fully exploited during the development of an image reconstruction algorithm for stroke diagnostics. In the same context, we formulated a forward problem to study the MW scattering behaviour of a 3D Zubal head model for brain stroke analysis. The solution of the subject MW scattering problem provided us with basic guidelines for the design of a real-time MW head-imaging system. It also helped us in determining an appropriate position for the dipole antenna array, a suitable frequency range and the permissible power levels. The computational domain around the Zubal head model was confined using perfectly matched layers. In addition, continuity boundary conditions were applied between the Zubal head model and air or different layers of brain tissues inside the head model using equation (3.3), where *E* denotes the electric field intensity (V m^−1^) and *n* is the normal vector. These settings realized the real-life environmental and operational conditions of an actual MW head-imaging system in the simulation platform. Figure 10 shows the simulation set-up of a MW head-imaging forward problem.

3.3 $$\text{n}\times ({\text{E}}_{1}-{\text{E}}_{2})=0.$$

In a head-imaging simulation set-up, the interaction between MW signals and a human head model is studied by estimating the E-Field values inside and around the head model using Maxwell's equations for EM waves. Depending upon the requirements of a study, the solution of a MW scattering forward problem is found out either in the time or frequency domain using an appropriate numerical method. Several numerical methods in practice for this purpose include MoM, FDTD, FEM and transmission line matrix [55,56]. In our 3D simulations, the values of transmitted and backscattered E-Field signals have been calculated using the Helmholtz vector wave equation form of Maxwell's equations, derived in a time-harmonic domain (equation (3.4)). This information will be used to develop an efficient image reconstruction algorithm for brain stroke analysis using the designed head-imaging system. In the equation, ∇ is the gradient operator, *μ_r_* is the relative permeability, *E* is the electric field intensity (V m^−1^), $k_{0}=\omega \sqrt{\mu_{0}\epsilon_{0}}$ is the free-space wavenumber (rad m^−1^), *ε_r_* is the relative permittivity, *σ* is the electrical conductivity (S m^−1^) and *ω* is the angular frequency (rad s^−1^).

3.4 $$\nabla \times \mu_{r}^{-1}(\nabla \times \text{E})-k_{0}^{2}\left(\varepsilon_{r}-\frac{j\sigma }{\omega \varepsilon_{0}}\right)\text{E}=0.$$

**Figure 11.**
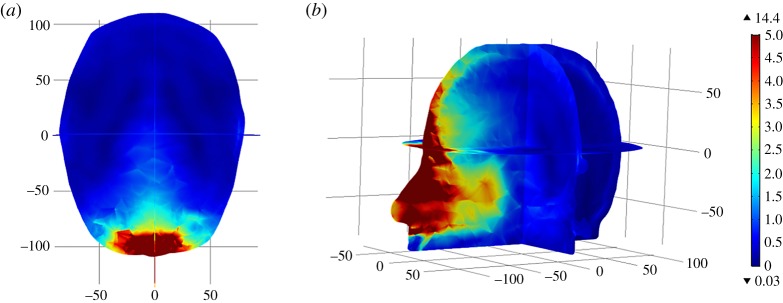
Spatial distribution of the E-Norm inside the 3D Zubal head model at the 1 GHz frequency using dipole antenna-7: (*a*) top view and (*b*) three-dimensional view.

In this study, we have selected the FEM numerical technique to perform a frequency-domain analysis of the simulation set-up for MW head imaging. The FEM is preferred because it exhibits least discretization error while modelling the complex geometries having anisotropic dielectric properties and immersed in a non-homogeneous background medium [22]. Therefore, the FEM is able to model the complex and inhomogeneous geometry of a 3D human head model in a more accurate manner. In addition, FEM deploys a differential equations framework, and hence is more suitable for conducting a steady-state analysis of the 3D human head MW scattering problem, once compared with other numerical techniques. Figure 11 shows the spatial distribution of the electric field norm (E-Norm) inside the 3D Zubal head model at the 1 GHz frequency using dipole antenna-7. In the figure, the colour bar maps E-Norm values (V m^−1^), with the red colour highlighting the locations of higher E-Norm values, and the blue colour indicating the lower ones. The FE mesh comprised 416 327 domain elements and the solution converged in 4 min and 30 s with a 2 672 334 number of degrees of freedom (DOF) using the Biconjugate Gradient Stabilized (BiCGStab) iterative solver with the multigrid option.

### 3.6. Finite-element mesh convergence

The FE analysis of a MW head-imaging set-up requires determining an appropriate mesh size in the simulation platform. The objective is to achieve reliable results with the least error count and minimum computational time, regardless of the mesh size or density. Therefore, a mesh convergence analysis was performed to figure out the most efficient FE mesh for solving the 3D Zubal head MW scattering problem. For a better comparison analysis, the mesh size was kept same for all three cases (normal, haemorrhagic and ischaemic). Therefore, for the normal brain case an emulated stroke sphere was assigned the dielectric properties of healthy brain tissues, accordingly. Different FE mesh models were generated following a criterion of at least five elements per wavelength (*λ*/5), to find out an accurately converged solution of the subject 3D MW scattering problem. A value of 1×10^−3^ error tolerance and the normal brain case were considered during the FE mesh convergence analysis. Simulations were executed on a high-performance workstation having an Intel Core i7-2600 processor (3.4 GHz, 4 Cores) and 16 GB RAM.

**Figure 12.**
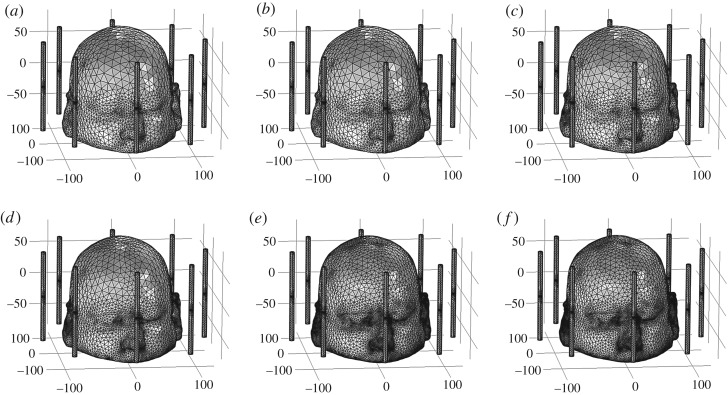
FE mesh mapping onto the 3D Zubal head model: (*a*) mesh model-1, (*b*) mesh model-2, (*c*) mesh model-3, (*d*) mesh model-4, (*e*) mesh model-5 and (*f*) mesh model-6.

**Figure 13.**
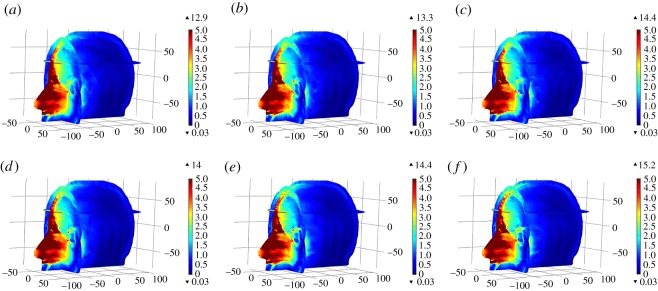
Spatial distribution of the E-Norm inside the 3D Zubal head model at the 1 GHz frequency using dipole antenna-7: (*a*) mesh model-1, (*b*) mesh model-2, (*c*) mesh model-3, (*d*) mesh model-4, (*e*) mesh model-5 and (*f*) mesh model-6.

To perform the FE mesh convergence analysis, we used a dipole antenna placed at location-7 and operating at the 1 GHz frequency with 1 V/ 75 Ω input values. The E-Norm evaluations were made at the centre of the stroke sphere, emulated at the front location-1 but filled with healthy brain tissues' dielectric properties. We used the BiCGStab iterative solver with the multigrid option to perform this analysis. The FE mesh model of six types with different levels of coarseness were generated for a 3D Zubal head model (table 3). In all FE models, the mesh parameters for an air region and the antenna array elements were fixed. However, the mesh size varied in the head region across different FE models because it is an investigation domain for brain stroke detection. Figure 12 maps each FE mesh model onto the Zubal head phantom and figure 13 displays the spatial distribution of the E-Norm inside the Zubal head phantom against each mesh model at 1 GHz.

**Table 3.** FE mesh models with element size parameters for the 3D Zubal head microwave imaging simulation set-up.

model no.	max. size (mm)	min. size (mm)	max. element growth rate	curvature factor	resolution of narrow regions
air region and antenna array					
	59.96	2.8	1.4	0.4	0.7
head region					
1	59.96	12.6	1.5	0.6	0.5
2	59.96	7	1.45	0.5	0.6
3	59.96	0.84	1.4	0.4	0.7
4	59.96	0.56	1.35	0.3	0.85
5	59.96	1.12	1.3	0.2	1
6	59.96	0.14	1.3	0.2	1

**Figure 14.**
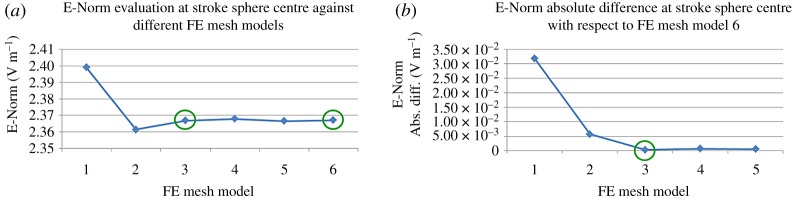
FE mesh convergence analysis for the 3D Zubal head model: (*a*) E-Norm evaluation at the stroke centre and (*b*) E-Norm absolute difference with respect to extremely fine mesh at the stroke centre.

The information about the number of DOF, the solution time and the E-Norm value at the centre of the stroke sphere is provided in table 4. During E-Norm value convergence analysis, it was determined that FE Model 3 provided a reliable solution within an error tolerance of 1×10^−3^, once compared to extremely fine FE Model 6 results. In addition, FE Model 3 comprised less than half the number of mesh elements and solved the subject 3D MW scattering problem efficiently in less time with a reduced number of DOF. Therefore, we preferred FE Model 3 over the finer FE Models (4, 5 and 6) and used it during our subsequent analysis of a MW head-imaging set-up for brain stroke diagnostics using a 3D Zubal head model. FE mesh convergence analysis for the Zubal head model, based on E-Norm and E-Norm absolute difference evaluation at the centre of the stroke sphere inserted at location-1, is shown in figure 14.

**Table 4.** FE solution information and E-Norm evaluation at the centre of the stroke sphere against different mesh models.

model no.	elements	d.f.	study time (min, s)	iterations	E-Norm (V m^−1^)	E-Norm abs. diff. (V m^−1^)
1	353 553	2 274 496	16 min, 12 s	31	2.3991	3.19 × 10^−02^
2	359 521	2 312 300	14 min, 18 s	24	2.3615	5.70 × 10^−03^
3	416 327	2 672 334	9 min, 17 s	5	2.3669	3.00 × 10^−04^
4	532 532	3 408 692	10 min, 4 s	3	2.3679	7.00 × 10^−04^
5	750 999	4 792 870	15 min, 51 s	4	2.3667	5.00 × 10^−04^
6	866 823	5 526 844	26 min, 32 s	4	2.3672	

### 3.7. Iterative solvers computational time

A time-efficient design analysis of a MW head-imaging system is performed through the solution time comparison of a human head MW scattering problem in the simulation platform. Therefore, in order to solve the 3D Zubal head MW scattering problem in a time-efficient manner, we performed a computational time comparison analysis between three types of iterative solver, namely generalized minimum residual (GMRES), flexible generalized minimum residual (FGMRES) and biconjugate gradient stabilized (BiCGStab) with the multigrid option. During the evaluation of each iterative solver, we used the FE mesh Model 3, a dipole antenna-7 operating at the 1 GHz frequency with 1 V/75 Ω input values and a stroke sphere inserted at the front location-1 but filled with healthy brain tissues’ dielectric properties. Table 5 provides the details of computational time, E-Norm evaluation at the stroke centre and the memory resources occupied by each iterative solver. It was observed that the GMRES method outperformed the other two iterative methods in terms of computational time. In addition, each iterative solver calculated the same E-Norm values and the memory resources requirement was comparable too. Therefore, we preferred the use of the GMRES iterative solver for finding the solution of the subject 3D human head MW scattering problem and presenting our brain stroke identification analysis in this study.

**Table 5.** Computational time and memory resources utilization of different iterative solvers for the 3D Zubal head microwave scattering problem.

solver type	solution time (min, s)	study time (min, s)	iterations	E-Norm (V m^−1^) stroke centre	phys. mem. (GB)	virtual mem. (GB)
GMRES	3 min, 39 s	8 min, 21 s	8	2.3669	11.23	14.24
FGMRES	4 min, 10 s	8 min, 52 s	15	2.367	12.13	15.07
BiCGStab	4 min, 30 s	9 min, 14 s	5	2.3669	11.05	13.99

## 4. Results and discussion

In this section, we present the simulation results of the MW scattering phenomenon demonstrated by a 3D Zubal head model. Two types of brain stroke at three different locations inside the head model were emulated to explain the changes in the E-Field pattern due to the presence of abnormal tissues' dielectric properties. A significant contrast in spatial distribution of the E-Field was also observed at an approximate location of the stroke, after comparison to the normal case. This information can be effectively used during the development of an image reconstruction algorithm for brain stroke localization and classification. The findings of mesh convergence and iterative solvers comparison analysis were fully exploited in this section to perform an error-free and time-efficient simulation analysis of a MW head-imaging system for brain stroke diagnostics. Later, a brief summary on the ionization effects of MW signals on brain tissues is provided to ensure the safety of the human head.

### 4.1. Electric field analysis for brain stroke detection

The E-Field distribution analysis of a 3D Zubal head model was performed for three cases (normal, haemorrhagic and ischaemic). The effects of an emulated stroke on the E-Field pattern were studied at three locations with different depths inside the head model (front, centre and sides). The absolute difference between electric field norm (E-Norm) values of the normal and stroke-affected tissues was used to localize and classify the types of brain stroke. A stroke inserted at the front location-1 and the side location-3 was investigated using dipole antennae 7 and 1, respectively. However, the effects of an emulated stroke at the centre location-2 were examined by comparing the simulation results of using either a dipole antenna 7 or 1. Each dipole antenna was placed at a distance of 3–4 cm from the side of the head model and transmitted MW signals at the 1 GHz frequency with 6.6 mW power. An FE mesh Model 3 and GMRES iterative solver were used during these MW head-imaging simulation analyses, due to the reasons discussed in the previous section.

#### 4.1.1. Brain stroke at front location-1

**Figure 15.**
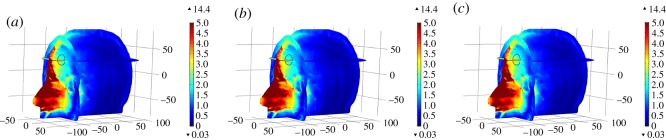
Spatial distribution of the E-Norm inside the 3D Zubal head model using stroke location-1 and dipole antenna-7 at the 1 GHz frequency: (*a*) normal brain, (*b*) haemorrhagic and (*c*) ischaemic.

The E-Field distribution maps for stroke analysis at the front location-1 (0 mm, −70 mm, 26.6 mm) were generated using a dipole antenna-7, facing the nose of the Zubal head model. In the case of a normal brain, an induced stroke sphere was filled with healthy brain tissues' dielectric properties. In comparison, the haemorrhagic and ischaemic stroke assumed (ε_r_ = 61.0650, *σ* = 1.5829 S m^−1^) and (ε_r_ = 30, *σ* = 0.5 S m^−1^) dielectric properties, respectively [33,34]. Figure 15 portrays the spatial distribution of E-Norm inside the 3D Zubal head model for three different cases at the 1 GHz frequency. The values of E-Norm and E-Norm absolute difference between the normal and stroke-affected brain tissues evaluated at seven points of a stroke sphere are provided in table 6. It was observed that the penetration of the E-Field is more progressive and uniform in the normal brain case, once compared to the stroke-affected brain. It is due to the fact that a brain stroke introduces an additional layer of tissues with different dielectric properties in the propagation path of MW signals, which causes more scattering effects.

**Table 6.** E-Norm and E-Norm absolute difference evaluation at stroke location-1 points inside the 3D Zubal head model using dipole antenna-7 at 1 GHz.

	evaluation point						
head model	left (V m^−1^)	front (V m^−1^)	bottom (V m^−1^)	centre (V m^−1^)	top (V m^−1^)	back (V m^−1^)	right (V m^−1^)
normal brain	2.0562	2.6091	2.3195	2.3669	1.7963	1.8414	1.8853
haemorrhagic	1.8877	2.4914	2.4588	2.2736	1.8274	1.8212	1.6098
ischaemic	2.4593	3.0701	2.2696	2.4753	1.7742	1.8901	2.2142
abs. diff. normal to haemorrhagic	0.1685	0.1177	0.1393	0.0933	0.0311	0.0202	0.2755
abs. diff. normal to ischaemic	0.4031	0.461	0.0499	0.1084	0.0221	0.0487	0.3289

**Figure 16.**
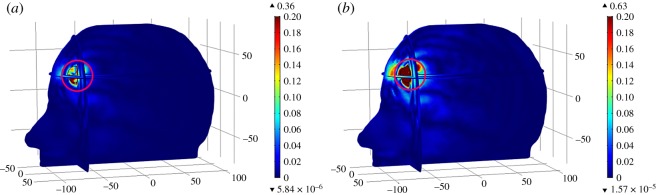
Spatial distribution of the E-Norm absolute difference between the normal and stroke-affected Zubal head models using stroke location-1 and dipole antenna-7 at the 1 GHz frequency: (*a*) haemorrhagic and (*b*) ischaemic.

The spatial distribution of E-Norm absolute difference between the normal and stroke-affected Zubal head models for two types of stroke at the 1 GHz frequency is shown in figure 16, where the colour bar represents E-Norm absolute difference (V m^−1^). In each case, the maximum E-Norm absolute difference value existed at an approximate location of brain stroke inside the Zubal head model (encircled red). The maximum differences were calculated as 0.36 V m^−1^ and 0.63 V m^−1^, respectively, for the hemorrhagic and ischemic affected head models. The difference values evaluated at stroke sphere points were greater in the case of ischemic stroke (0.461 V m^−1^) once compared with the haemorrhagic stroke (0.2755 V m^−1^). Large differences were observed in an ischaemic stroke case because its dielectric properties contrast considerably with respect to GM tissues (ε_r_ = 52.282, *σ* = 0.98541 S m^−1^) and also the major part of the stroke was emulated in the GM region. By contrast, the haemorrhagic stroke dielectric properties were much closer to GM tissues, therefore it demonstrated less deviation from the MW scattering behaviour of the normal case, compared to an ischaemic stroke case.

#### 4.1.2. Brain stroke at centre location-2

**Figure 17.**
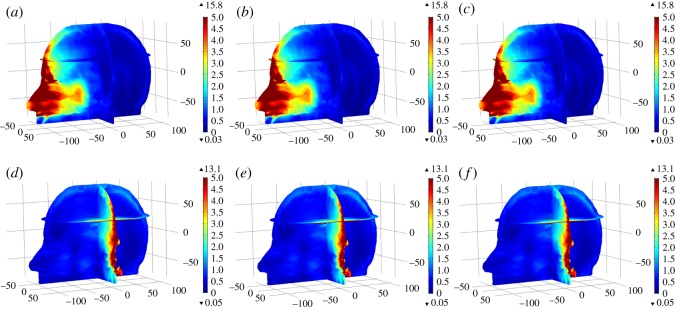
Spatial distribution of the E-Norm inside the 3D Zubal head model using stroke location-2 at the 1 GHz frequency: (*a*) normal brain and AE-7, (*b*) haemorrhagic and AE-7, (*c*) ischaemic and AE-7, (*d*) normal brain and AE-1, (*e*) haemorrhagic and AE-1, and (*f*) ischaemic and AE-1.

**Figure 18.**
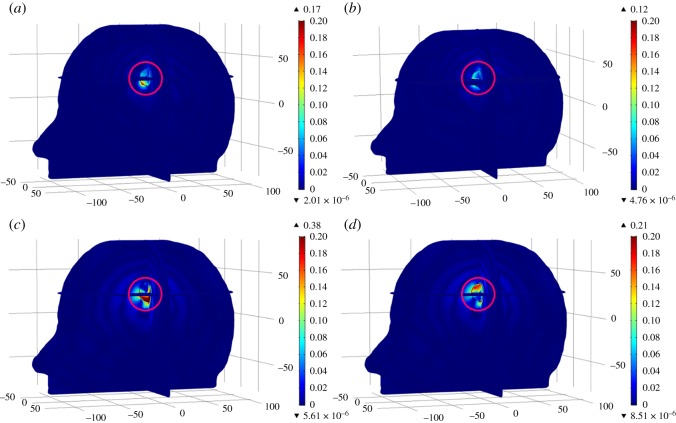
Spatial distribution of the E-Norm absolute difference between the normal and stroke-affected Zubal head models using stroke location-2 at the 1 GHz frequency: (*a*) haemorrhagic and AE-7, (*b*) ischaemic and AE-7, (*c*) haemorrhagic and AE-1, and (*d*) ischaemic and AE-1.

The E-Field distribution analysis for a brain stroke emulated deep at the centre location-2 (0 mm, 0 mm, 26.6 mm) was performed using either a dipole antenna (AE) 7 or 1. At this location, the stroke sphere overlapped both the WM and GM regions. The spatial distribution of the E-Norm inside the 3D Zubal head model for all cases at the 1 GHz frequency is shown in figure 17. The values of E-Norm and E-Norm absolute difference evaluated at seven points of a stroke sphere, using either a dipole antenna 7 or 1, are provided in table 7. In comparison with dipole antenna-7, a dipole antenna-1 placed on the right side of the Zubal head model was closer to the centre location-2 of an induced brain stroke. Therefore, high values of E-Norm and E-Norm absolute difference have been observed while using a dipole antenna-1 during brain stroke analysis at the centre location-2. The spatial distribution of E-Norm absolute difference between the normal and stroke-affected Zubal head models for two types of stroke, using either a dipole antenna 7 or 1, is mapped at 1 GHz in figure 18.

**Table 7.** E-Norm and E-Norm absolute difference evaluation at stroke location-2 points inside the 3D Zubal head model using dipole antennae 7 and 1 at 1 GHz.

		evaluation point						
head model	source	left (V m^−1^)	front (V m^−1^)	bottom (V m^−1^)	centre (V m^−1^)	top (V m^−1^)	back (V m^−1^)	right (V m^−1^)
normal brain	dipole	0.43842	0.61663	0.5317	0.47691	0.5078	0.32867	0.33025
haemorrhagic	AE-7	0.35991	0.59957	0.46568	0.45132	0.46774	0.35526	0.2691
ischaemic		0.49262	0.67952	0.52036	0.50464	0.56583	0.28745	0.38153
abs. diff. normal to haemorrhagic		0.07851	0.01706	0.06602	0.02559	0.04006	0.02659	0.06115
abs. diff. normal to ischaemic		0.0542	0.06289	0.01134	0.02773	0.05803	0.04122	0.05128
normal brain	dipole	0.74138	0.81669	1.1244	0.87118	0.65897	0.79862	1.1648
haemorrhagic	AE-1	0.77422	0.70933	1.0414	0.84544	0.67824	0.71786	1.0399
ischaemic		0.62812	0.95186	1.0954	0.94822	0.74071	0.91665	1.2552
abs. diff. normal to haemorrhagic		0.03284	0.10736	0.083	0.02574	0.01927	0.08076	0.1249
abs. diff. normal to ischaemic		0.11326	0.13517	0.029	0.07704	0.08174	0.11803	0.0904

Similar to case-1, the maximum E-Norm absolute difference values were observed at an approximate location of brain stroke inside the Zubal head model (encircled red). Using a dipole antenna-7, the maximum difference values for the haemorrhagic and ischaemic head models were measured as 0.17 V m^−1^ and 0.12 V m^−1^, respectively. In comparison, these differences were accordingly found to be 0.38 V m^−1^ and 0.21 V m^−1^ using a dipole antenna-1. It is noteworthy that using either of the dipole antennae, the maximum difference value was greater in a haemorrhagic stroke case, once compared with an ischaemic stroke. This is due to the fact that a slightly bigger part of the stroke sphere was intersecting the WM region and the dielectric properties of the haemorrhagic stroke differ significantly with respect to WM tissues (ε_r_ = 38.577, *σ* = 0.6219 S m^−1^). These difference values were comparable using a dipole antenna-7; however, the differences became evident once a dipole antenna-1, closer to the centre location-2 of an induced brain stroke, was used.

#### 4.1.3. Brain stroke at side location-3

**Figure 19.**
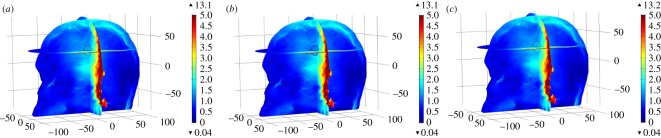
Spatial distribution of the E-Norm inside the 3D Zubal head model using stroke location-3 and dipole antenna-1 at the 1 GHz frequency: (*a*) normal brain, (*b*) haemorrhagic and (*c*) ischaemic.

**Figure 20.**
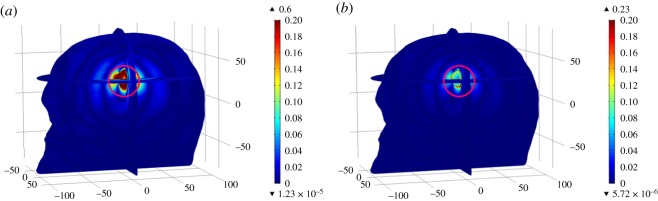
Spatial distribution of the E-Norm absolute difference between the normal and stroke-affected Zubal head models using stroke location-3 and dipole antenna-1 at the 1 GHz frequency: (*a*) haemorrhagic and (*b*) ischaemic.

The E-Field distribution of a brain stroke emulated at the side location-3 (22 mm, 0 mm, 26.6 mm) was analysed using a dipole antenna-1, facing the right side of the Zubal head model. Figure 19 displays the spatial distribution of the E-Norm inside the 3D Zubal head model for three different cases at the 1 GHz frequency. Table 8 provides the values of E-Norm and E-Norm absolute difference evaluated at seven points of a stroke sphere, induced at the side location-3 and using a dipole antenna-1. Figure 20 portrays the spatial distribution of E-Norm absolute difference between the normal and stroke-affected Zubal head models for two types of stroke at 1 GHz. Similar to the two cases above, the maximum E-Norm absolute difference values were measured at an approximate location of the brain stroke inside the Zubal head model (encircled red). The maximum differences were calculated as 0.60 V m^−1^ and 0.23 V m^−1^, respectively, for the haemorrhagic and ischaemic head models. The difference values evaluated at stroke sphere points were greater in the case of haemorrhagic stroke (0.296 V m^−1^) once compared with an ischaemic stroke (0.1241 V m^−1^). Large differences were observed in a haemorrhagic stroke case because its dielectric properties contrast reasonably with respect to WM tissues (ε_r_ = 38.577, *σ* = 0.6219 S m^−1^), and also the major part of stroke was emulated in the WM region. In contrast, the ischaemic stroke dielectric properties were much closer to WM tissues, therefore it exhibited less deviation from the MW scattering behaviour of the normal case, compared to a haemorrhagic stroke case.

**Table 8.** E-Norm and E-Norm absolute difference evaluation at stroke location-3 points inside the 3D Zubal head model using dipole antenna-1 at 1 GHz.

	evaluation point						
head model	left (V m^−1^)	front (V m^−1^)	bottom (V m^−1^)	centre (V m^−1^)	top (V m^−1^)	back (V m^−1^)	right (V m^−1^)
normal brain	1.2393	1.3968	1.77	1.5188	1.3552	1.4287	1.8078
haemorrhagic	1.2246	1.1008	1.938	1.3899	1.3801	1.1676	1.5984
ischaemic	1.1803	1.5209	1.7152	1.566	1.3474	1.5366	1.9313
abs. diff. normal to haemorrhagic	0.0147	0.296	0.168	0.1289	0.0249	0.2611	0.2094
abs. diff. normal to ischaemic	0.059	0.1241	0.0548	0.0472	0.0078	0.1079	0.1235

Through 3D EM simulations, we have demonstrated the MW scattering phenomenon exhibited by the normal and stroke-affected Zubal head model in a MW head-imaging set-up. A significant contrast in E-Field values has been observed at an approximate location of the brain stroke with respect to the normal case. These differences vary with the type and location of the brain stroke emulated in a head model. These results assure the feasibility of a human head MW imaging system for brain stroke diagnostics. We also compared our MW simulation results with recent research studies conducted on the 3D Zubal head phantom [15,35,36,47–49]. These studies implemented different numerical techniques and tissue-mapping schemes. It is worth mentioning that our results were in good agreement with previous studies' concluding remarks. However, we preferred the use of the FEM over FDTD or MoM to time-efficiently solve the subject MW scattering problem in the frequency domain and model the complex geometry of a 3D human head with least discretization error. In addition, our 3D anthropomorphic head model was anatomically more realistic and structurally detailed. This has not been available in the literature to date.

Summarizing, only a few studies in the MW head-imaging area have presented a comparison analysis between both types of brain stroke and that was limited. Some studies deployed a geometrically simplified head model [27] or a rudimentary head phantom [42], whereas some followed a radar approach [57] or developed a statistical classifier algorithm [58]. In our study, we have presented a brain stroke detection and differentiation analysis involving our anatomically more realistic 3D head model. The anthropomorphic head model was generated using our novel tissue-mapping scheme and a mixed-model approach. In comparison, we implemented the FEM technique and the GMRES iterative solver to perform a frequency-domain analysis of the subject 3D MW scattering problem in an error-free and time-efficient manner. Moreover, our brain stroke comparison analyses were based on a tomographic approach.

The information of transmitted and scattered E-Field signals, obtained from the solution of a MW scattering forward problem, can effectively be exploited during the development of an efficient image reconstruction algorithm. The authors also believe they can implement it in their future research. An image reconstruction algorithm involves the construction of reliable head images for brain stroke localization and classification. In fact, it is required to find out the solution of an inverse scattering problem, which is mathematically an ill-posed and nonlinear problem. In a MWT approach, high-quality brain images are created by mapping the dielectric properties of brain tissues to indicate the location of anomalies. In real-life applications, an image reconstruction algorithm calculates the dielectric properties of brain tissues from scattered (*S*)-parameters information only. Thereafter, generating high-resolution brain images can help identify the location and types of brain stroke by detecting the presence of abnormal tissues' dielectric properties.

In MW head tomography, multiple inversion schemes are being used including Gauss-Newton inversion (GNI), conjugate-gradient inversion (CGI), Born iterative method (BIM) and contrast source inversion (CSI) [22,59–63]. In addition, different regularization techniques are also implemented as an add-on to these inversion methods. This results in image quality enhancement and robustness to noise at the cost of additional memory and processing time. In our future studies, we will analyse the performance of these inversion schemes by evaluating their accuracy parameters and computational resource requirement through a detailed literature survey. Thereafter, we will use the simulation results of our present study to develop a more accurate and efficient image reconstruction algorithm, suitable for MW head tomography applications. A frequency-hopping technique will be considered using our developed 3D frequency-dispersive head model to construct high-quality brain images in a recursive manner [14,61,63]. Additionally, the requirement to add a suitable coupling medium around the head model and redesign the antenna array will also be taken into account.

### 4.2. Specific absorption rate analysis

Specific absorption rate (SAR) analysis is performed to ensure the safe exposure of EM signals to human body tissues. A SAR value quantifies the amount of radiation absorbed by human body tissues and the resultant temperature increase under the direct exposure to EM signals. In previous research studies, SAR values for human head tissues were estimated mainly using a mobile phone placed adjacent to the head model. Most of these studies presented computer simulations involving a numerical head model [64–68], while some were based on theoretical analysis [69]. The SAR value is defined as the amount of power dissipated per unit mass, calculated in watts per kilogram (W kg^−1^) using equation (4.1), where *σ* is the tissue electrical conductivity ( S m^−1^), |*E|* is the electric field norm (V m^−1^) and *ρ* is the tissue density (kg m^−3^).

4.1 $$\text{SAR}=\frac{\sigma |\text{E}{|}^{2}}{2\rho }.$$

**Figure 21.**
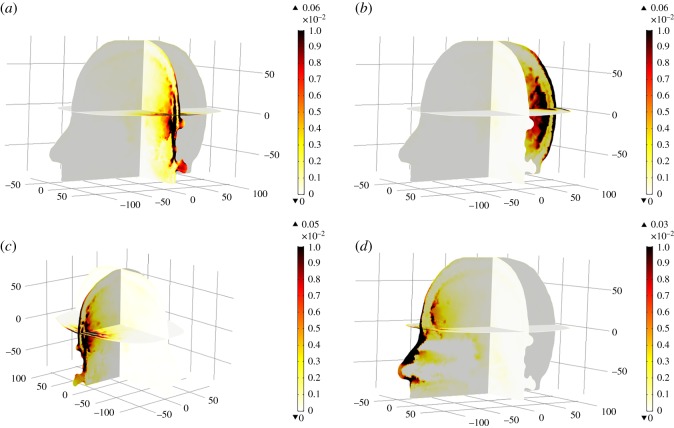
Spatial distribution of SAR values inside the 3D Zubal head model at 1 GHz: (*a*) dipole AE-1, (*b*) dipole AE-3, (*c*) dipole AE-5 and (*d*) dipole AE-7.

According to the IEEE standard for safety levels (C95.1-2005) [70] and ICNIRP safety guidelines for limiting exposure to EM fields [71], an average SAR value below 2 W kg^−1^ over 10 g of tissue is reported as a safety limit. To examine the ionization effects of MW signals to a human head, we considered four locations of a dipole antenna (1, 3, 5 and 7) and an average tissue density of 1050 kg m^−3^ in our MW head-imaging simulations. Each dipole antenna was placed at a 3–4 cm distance from the side of a 3D Zubal head model and operated in a sequential mode at the 1 GHz frequency with 6.6 mW transmitted power. Figure 21 portrays the spatial distribution of SAR inside the 3D Zubal head model at 1 GHz for four different cases. In the figure, the thermal colour bar maps local SAR values (W Kg^−1^), with the dark brown colour highlighting the locations of higher SAR values, while the white colour indicates the lower ones.

In all cases, the maximum local SAR value was calculated to be in the range 0.03–0.06 W kg^−1^ and existed at the skin layer. It is highlighted that these values are well within the safety criteria and depend a lot on the conductivity of brain tissues. In addition, our results are also comparable with an earlier study conducted on a 3D Zubal head phantom [35]. The authors used a directional antenna transmitting at 1 mW power. The peak SAR values of 0.016 W kg^−1^ and 0.006 W kg^−1^ were reported at the 1.8 GHz and 1.1 GHz frequencies, respectively. In our simulations, slightly higher SAR values (0.03–0.06 W kg^−1^) were observed at the 1 GHz frequency because the dipole antenna was transmitting at 6.6 mW power. However, these values are far below the suggested limit, thus guaranteeing the safe usage of a MW head-imaging system.

## 5. Conclusion

This study provides a deep insight into the application of the MWI technique for brain stroke diagnostics. Various aspects of the MW scattering phenomenon exhibited by a 3D anthropomorphic human head model were explored. Simulation results of this study suggested that MW head imaging may precisely indicate the location of a brain stroke by using the difference between E-Field values of the normal and stroke-affected tissues in an image reconstruction algorithm. Comparison analysis highlighted that these differences vary with the type and location of an induced brain stroke. FE mesh convergence and iterative solvers computational time analyses were performed to achieve error-free results in a time-efficient manner. In addition, SAR analysis ensured the safety of using MW signals in the design of human head diagnostic systems. The main contribution of this study is summarized below:-

(i) A novel tissue-mapping scheme using a mixed-model approach has been implemented to generate an anatomically more realistic 3D human head model. This in turn provides a full brain head model having frequency-dependent dielectric properties of tissues, with prospective utilization in multiple biomedical applications.
(ii) FEM numerical technique is applied for the first time to accurately model a 3D realistic head geometry with least discretization error and to perform a steady-state analysis of the MW scattering phenomenon demonstrated by a 3D human head model.
(iii) A MW scattering comparison analysis between both types of brain stroke at different locations inside the head model has been efficiently performed in less time using the GMRES iterative solver.

In future, we will use the simulation results of our present 3D MW scattering problem to develop an efficient image reconstruction algorithm. Multiple inversion schemes will be analysed to construct good quality brain images in an accurate and time-efficient manner. Real-life noise effects will be introduced into the simulated E-Field database and accordingly the inverse algorithm will be incorporated with a suitable noise cancellation technique to nullify these effects. A multi-frequency swept approach will be implemented to generate high-resolution brain images in a recursive fashion by making use of our developed 3D frequency-dispersive head model. The possibility of using parallel processing techniques during the multi-source multi-frequency implementation will also be considered while finding out the solutions of MW head-imaging forward and inverse problems. Also, the optimal design parameters for the development of a MW head-imaging system for brain stroke diagnostics will be suggested.

## Supplementary Material

Zubal Head Phantom Tissue Mapping Scheme and Model Assignment

## Supplementary Material

4th Order Debye and Cole-Cole Models Parameters and Evaluation

## Supplementary Material

Matlab programming file for Zubal Head Phantom Frequency-Dispersive Properties Assignment

## Data accessibility

The datasets supporting this article have been uploaded as part of the electronic supplementary material. The additional data files are available at the public repository link http://doi.org/10.5061/dryad.bh39282.
