# Supplementary material for: Finite-element analysis of microwave scattering from a three-dimensional human head model for brain stroke detection
Source: R Soc Open Sci. 2018 Jul 11;5(7):180319. doi: 10.1098/rsos.180319 (PMC6083670; doi:10.1098/rsos.180319)
Supplement: 4th Order Debye and Cole-Cole Models Parameters and Evaluation [file rsos180319supp2.docx]

**FOURTH-ORDER DEBYE MODEL PARAMETERS**

| **Type** | **ϵ_inf** | **Δϵ1** | **Δϵ2** | **Δϵ3** | **Δϵ4** | **τ1** | **τ2** | **τ3** | **τ4** | **σ_s** |
| --- | --- | --- | --- | --- | --- | --- | --- | --- | --- | --- |
| Free Space | 1 | 0 | 0 | 0 | 0 | 0 | 0 | 0 | 0 | 0; |
| Skin Dry | 6.5988 | 23.6622 | 9.4543 | 29.7866 | 4.1333e+04 | 4.1221e-12 | 3.5703e-11 | 1.3321e-09 | 1.0752e-06 | 0.0242; |
| Bone Cortical (Skull) | 1.0000 | 10.3665 | 2.0000 | 5.7498 | 1.0994e+03 | 1.0000e-11 | 1.2664e-10 | 1.3040e-09 | 6.1201e-06 | 0.0527; |
| Blood | 2.5299 | 42.1250 | 11.3375 | 703.1670 | 5.0130e+03 | 4.2278e-12 | 3.6241e-11 | 9.6392e-09 | 1.0028e-06 | 0.9998; |
| White Matter | 1.0000 | 33.0086 | 5.9024 | 45.6450 | 1.0280e+03 | 8.2606e-12 | 1.2737e-10 | 1.7947e-09 | 2.1134e-05 | 0.2686; |
| Fat | 2.1647 | 2.3553 | 1.5130 | 16.8063 | 1.2582e+03 | 7.3855e-12 | 6.6691e-10 | 8.7830e-09 | 2.8814e-06 | 0.0076; |
| Skin Wet | 2.26 | 33.493 | 11.539 | 14.2 | 61.365e3 | 4.618e-12 | 36.7e-12 | 0.553e-9 | 1.301e-6 | 109.2e-3 |
| Eye Tissue (Sclera) | 3.9114 | 48.3026 | 6.7413 | 1.1459e+03 | 6.9026e+04 | 1.0000e-11 | 2.0890e-10 | 1.6345e-08 | 2.2689e-06 | 0.0025; |
| CSF | 1.1364 | 48.5481 | 18.6916 | 3.0552e+03 | 3.5094e+04 | 2.4988e-12 | 2.0008e-11 | 2.7630e-08 | 1.0006e-06 | 0.9997; |
| Dura | 2.1639 | 42.4724 | 4.9144 | 16.5288 | 2.1286e+03 | 9.4680e-12 | 1.2211e-10 | 9.7133e-10 | 7.4308e-05 | 0.5998; |
| Cerebellum | 2.5017 | 44.9855 | 4.7681 | 327.7204 | 6.2895e+03 | 8.7388e-12 | 2.1397e-10 | 4.5307e-09 | 1.0609e-06 | 0.1406; |
| Bone Marrow | 1.8415 | 2.8863 | 1.0000 | 75.1316 | 160.2949 | 7.2673e-12 | 1.9290e-10 | 2.8080e-08 | 0.0891 | 1.0139e-04; |
| Muscle Transverse | 1.8608 | 51.198 | 9.646 | 33.748 | 8.4595E4 | 7.921E-12 | 9.761E-11 | 1.477E-9 | 1.2564E-6 | 0.1138; |
| Spinal Cord | 2.0909 | 29.6301 | 4.0300 | 97.8208 | 575.9411 | 8.8234e-12 | 2.2977e-10 | 3.2026e-09 | 0.0984 | 0.1664; |
| Gray Matter | 1.3728 | 16.6423 | 31.4435 | 59.8685 | 1.1799e+04 | 1.0000e-13 | 2.1513e-11 | 1.3036e-09 | 1.1273e-06 | 0.3655; |
| Cartilage | 1 | 36.1150 | 7.3201 | 426.4864 | 2.8086e+04 | 1.0000e-11 | 1.8491e-10 | 7.2579e-09 | 5.9221e-05 | 0.0223; |
| Muscle Parallel | 1.8608 | 51.198 | 9.646 | 33.748 | 8.4595E4 | 7.921E-12 | 9.761E-11 | 1.477E-9 | 1.2564E-6 | 0.1138 |
| Bone Cancellous (Spongy) | 1.428 | 16.045 | 3.918 | 25.65 | 501.4e2 | 9.154e-12 | 245.1e-12 | 2.152e-9 | 7.04e-6 | 9.3e-3; |

**FOURTH-ORDER COLE-COLE MODEL PARAMETERS**

| **Type** | **ϵ_inf** | **Δϵ1** | **τ1** | **α1** | **Δϵ2** | **τ2** | **α2** | **Δϵ3** | **τ3** | **α3** | **Δϵ4** | **τ4** | **α4** | **σ_s** |
| --- | --- | --- | --- | --- | --- | --- | --- | --- | --- | --- | --- | --- | --- | --- |
| Stomach | 4.000 | 60.00 | 7.958 | 0.100 | 2000 | 79.577 | 0.100 | 1.00e+5 | 159.155 | 0.200 | 4.00e+7 | 15.915 | 0.000 | 0.500 |
| Tongue | 4.000 | 50.00 | 7.958 | 0.100 | 4000 | 159.155 | 0.100 | 1.00e+5 | 159.155 | 0.200 | 4.00e+7 | 15.915 | 0.000 | 0.250 |
| Trachea | 2.500 | 38.00 | 7.958 | 0.100 | 400 | 63.662 | 0.100 | 5.00e+4 | 15.915 | 0.200 | 1.00e+6 | 15.915 | 0.000 | 0.300 |
| Eye tissue  (Lense-Nucleus) | 3.000 | 32.00 | 8.842 | 0.100 | 100 | 10.610 | 0.200 | 1.00e+3 | 15.915 | 0.200 | 5.00e+3 | 15.915 | 0.000 | 0.200 |
| Eye tissue  (Lense-Cortex) | 4.000 | 42.00 | 7.958 | 0.100 | 1500 | 79.577 | 0.100 | 2.00e+5 | 159.155 | 0.100 | 4.00e+7 | 15.915 | 0.000 | 0.300 |

**4^th^ Order Debye and Cole-Cole Models Evaluated at 1 GHz:**

| **Property** | **Value** |
| --- | --- |
| eps_complex_4_debye = | 1 |
| eps_rel_freespace_4_debye = | 1 |
| sigma_freespace_4_debye = | 0 |
| eps_complex_4_debye = | 39.6666 -12.6937i |
| eps_rel_skin_dry_4_debye = | 39.6666 |
| sigma_skin_dry_4_debye = | 0.7062 |
| eps_complex_4_debye = | 47.7684 -16.7415i |
| eps_rel_skin_wet_4_debye = | 47.7684 |
| sigma_skin_wet_4_debye = | 0.9314 |
| eps_complex_4_debye = | 4.6020 - 0.9612i |
| eps_rel_fat_4_debye = | 4.6020 |
| sigma_fat_4_debye = | 0.0535 |
| eps_complex_4_debye = | 12.6348 - 3.2906i |
| eps_rel_bone_cortical_4_debye = | 12.6348 |
| sigma_bone_cortical_4_debye = | 0.1831 |
| eps_complex_4_debye = | 18.7217 - 5.8967i |
| eps_rel_bone_cancellous_4_debye = | 18.7217 |
| sigma_bone_cancellous_4_debye = | 0.3280 |
| eps_complex_4_debye = | 37.8741 -13.4403i |
| eps_rel_white_matter_4_debye = | 37.8741 |
| sigma_white_matter_4_debye = | 0.7477 |
| eps_complex_4_debye = | 49.7739 -19.6222i |
| eps_rel_gray_matter_4_debye = | 49.7739 |
| sigma_gray_matter_4_debye = | 1.0916 |
| eps_complex_4_debye = | 55.5956 -33.9471i |
| eps_rel_blood_4_debye = | 55.5956 |
| sigma_blood_4_debye = | 1.8885 |
| eps_complex_4_debye = | 68.1756 -44.2254i |
| eps_rel_csf_4_debye = | 68.1756 |
| sigma_csf_4_debye = | 2.4603 |
| eps_complex_4_debye = | 48.0121 -18.3149i |
| eps_rel_dura_4_debye = | 48.0121 |
| sigma_dura_4_debye = | 1.0189 |
| eps_complex_4_debye = | 5.1292 - 1.0501i |
| eps_rel_bonemarrow_4_debye = | 5.1292 |
| sigma_bonemarrow_4_debye = | 0.0584 |
| eps_complex_4_debye = | 49.4544 -19.7148i |
| eps_rel_cerebellum_4_debye = | 49.4544 |
| sigma_cerebellum_4_debye = | 1.0968 |
| eps_complex_4_debye = | 33.1778 -11.3644i |
| eps_rel_spinal_cord_4_debye = | 33.1778 |
| sigma_spinal_cord_4_debye = | 0.6322 |
| eps_complex_4_debye = | 54.6089 -22.3164i |
| eps_rel_eye_tissue_4_debye = | 54.6089 |
| sigma_eye_tissue_4_debye = | 1.2415 |
| eps_complex_4_debye = | 40.2931 -15.7036i |
| eps_rel_cartilage_4_debye = | 40.2931 |
| sigma_cartilage_4_debye = | 0.8736 |
| eps_complex_4_debye = | 60.3305 -23.1972i |
| eps_rel_muscle_parallel_4_debye = | 60.3305 |
| sigma_muscle_parallel_4_debye = | 1.2905 |
| eps_complex_4_Cole = | 64.7973 -22.1394i |
| eps_rel_stomach_4_Cole = | 64.7973 |
| sigma_stomach_4_Cole = | 1.2316 |
| eps_complex_4_Cole = | 55.0170 -17.5274i |
| eps_rel_tongue_4_Cole = | 55.0170 |
| sigma_tongue_4_Cole = | 0.9751 |
| eps_complex_4_Cole = | 41.7785 -14.4221i |
| eps_rel_trachea_4_Cole = | 41.7785 |
| sigma_trachea_4_Cole = | 0.8023 |
| eps_complex_4_Cole = | 35.6667 - 9.2003i |
| eps_rel_eye_nucleus_4_Cole = | 35.6667 |
| sigma_eye_nucleus_4_Cole = | 0.5118 |
| eps_complex_4_Cole = | 46.3994 -14.8175i |
| eps_rel_eye_cortex_4_Cole = | 46.3994 |
| sigma_eye_cortex_4_Cole = | 0.8243 |
